# Electrophysiological Characterization of Neuropathy Complicating Type 1 Diabetes Mellitus

**DOI:** 10.1155/2019/2435261

**Published:** 2019-07-02

**Authors:** Nimat Abuelwafaa, Hana Ahmed, Ilham Omer, Mohamed Abdullah, Ammar Ahmed, Afraa Musa

**Affiliations:** ^1^Department of Physiology, Faculty of Medicine, University of Khartoum, 11111, Sudan; ^2^Department of Paediatrics and Child Health, Faculty of Medicine, University of Khartoum, 11111, Sudan

## Abstract

Diabetic peripheral neuropathy (DPN) involves sensory and motor nerves, resulting in demyelination as well as axonal degeneration. This study was conducted to describe the pattern of lower limb nerve involvement in children with type 1 diabetes mellitus (DM) based on the parameters of nerve conduction study (NCS). This cross-sectional study recruited 50 children with type 1 DM having mean disease duration of 4.92 ± 3.84 years who attended the referred clinic in Sudan Childhood Diabetes Center. Their mean age was 15.00 ± 2.19 years, 42% were males, and 58% were females. Twenty six matched healthy control subjects were involved; their mean age was 13.88 ± 2.46 years, 38.46% were males, and 61.54% were females. Bilateral NCS of the sensory and motor lower limb nerves was performed using Medelec Synergy machine. Interpretation of the patients' results was based on our own control reference values. Data was analysed using IBM SPSS statistics. Out of the 50 diabetic patients, 44 (88%) had electrophysiological evidence of peripheral neuropathy (abnormalities in at least two of the electrophysiological parameters). The majority (68.2%) had motor involvement and 31.8% had sensorimotor, while none of them (0%) had pure sensory involvement. Regarding abnormal NCS parameters (conduction velocity vs. amplitude of the compound action potential), conduction slowing feature predominated in 61.4% and only few (6.8%) showed amplitude reduction, while 31.8% showed mixed features. The most frequently affected nerve was the common peroneal, followed by posterior tibial, and the least was the sural nerve. The most sensitive parameter was the common peroneal conduction velocity. Motor precedes sensory nerve involvement. The most frequent neurophysiological abnormality was the conduction slowing, and the common peroneal was the most vulnerable nerve. These findings signify generation of a protocol for early screening of neuropathy in children with type 1 diabetes.

## 1. Background

Diabetes mellitus (DM) is considered as a group of disorders of heterogeneous etiology, characterized by chronic hyperglycemia and other metabolic abnormalities caused by defects in insulin secretion, insulin action, or both. Following a long duration of diabetes, microvascular complications (retinopathy, nephropathy, and neuropathy) and macrovascular complications (arteriosclerosis) occur [1]. Different parts of the nervous system are affected by diabetic neuropathies, therefore presented with diverse clinical manifestations. Chronic sensorimotor distal symmetric polyneuropathy and autonomic neuropathies are among the most common nerve insults of diabetes. Internationally, diabetic peripheral neuropathy (DPN) is defined as “the presence of symptoms and/or signs of peripheral nerve dysfunction in people with diabetes after the exclusion of other causes” [2]. DPN complicates both type 1 and type 2 DM. Distal sensory neuropathy can be classified into three: pure small fiber, mixed small and large fiber, and pure large fiber neuropathies [3]. In contrast to adults, children and adolescents often show minimal signs or symptoms of neuropathy early on in their disease; therefore, clinical examination is less sensitive and specific than nerve conduction studies (NCSs), which serves as the gold standard test in detection of subclinical neuropathy [4–8].

NCSs, the most informative electrodiagnostic tests, are noninvasive, standardized, and objective tests for measuring the dysfunction of large myelinated sensory and motor nerve fibers. They are included as an integral part of the case definition of polyneuropathy [9]. Neuropathy, whether demyelinating or axonal, can be determined on the basis of nerve conduction studies [10]. Demyelinating neuropathy is diagnosed when there is prolongation in latency and slowing in conduction velocity greater than 40% of the normal mean while the amount of axonal degeneration can be gauged by the degree of reduction in amplitudes of sensory or motor compound action potential (SNAP or CMAP) to distal stimulation [11]. NCS provides a sensitive but nonspecific index on the onset of DPN and is a valuable tool in detecting subclinical cases [12]. The concept of a subclinical or an asymptomatic form of neuropathy is well established [13]. The progression of neuropathy is assumed to be a continuum from normal nerve function to subclinical neuropathy detectable with electrophysiological tests to clinically evident neuropathy recognizable on neurological examination [13]. Using Dyck et al.'s criteria [14], subclinical DNP was defined by many researchers as the finding of changes in NCS in at least two nerves [15]. The most accurate diagnosis of distal symmetric polyneuropathy is better achieved through the combination of neuropathic symptoms, signs, and electrodiagnostic findings [9, 16, 17].

Diabetic neuropathy is caused by an interaction of the patient's susceptibility, vascular, metabolic, and environmental components. Many studies pointed to the risk factors for diabetic neuropathy, such as poor glycemic control, long duration of diabetes, older age of onset, male gender, height, alcohol use, hypertension, nicotine use, and hyperlipidemia [13, 18–21].

Although there has been considerable research of peripheral neuropathy in type 1 diabetes, still there is controversy regarding the pattern of nerve involvement in diabetic neuropathy. Early detection of diabetic neuropathy during childhood, using nerve conduction study as a screening tool, would allow timely intervention, with the possibility of reducing or delaying the incidence and progression of neuropathy and its consequences later in life. NCS results provide information about the severity of nerve involvement and help in the prediction of prognosis and response to strict glycemic control. In addition, detection of the pattern of nerve involvement aids in establishing a protocol for screening of DPN in its subclinical stage.

The aim of this study was to describe the pattern of nerve involvement in children with type 1 DM using nerve conduction studies. This was achieved by identifying the type of nerve involvement in relation to its function (motor, sensory, or sensorimotor), quantifying the frequency of the affected lower limb nerves (sural, common peroneal, and posterior tibial), and lastly differentiating the principal pathological pattern of nerve involvement (demyelinating or axonal degeneration) based on NCS findings (conduction velocity versus amplitude reduction).

## 2. Methods

This cross-sectional analytical study was conducted at the Department of Physiology in the Faculty of Medicine of Khartoum University. Fifty children with type 1 diabetes (29 females and 21 males), whose ages ranged from 10 to 18 years, were selected randomly from those attending the referred clinic in Sudan Childhood Diabetes Center from July to October 2016. Twenty-six healthy children (16 females and 10 males), age, weight, and height matched to the patient group, were selected randomly from Khartoum State population to serve as a control group for patients. Children suffering from peripheral neuropathy caused by other diseases (e.g., nutritional deficiency, infective causes, connective tissue disease, or drugs), or with a family history of hereditary neuropathy (e.g., Charcot–Marie–Tooth), were excluded from the study. The collected data included demographic data, relevant history including symptoms of neuropathy, complete neurological examination, HBA1c, and detailed NCS test results. The machine used was digital Medelec Synergy, which automatically measures the NCS parameters (amplitude and latency) of the SNAP or CMAP and calculates the conduction velocity (CV) in meter/second. Bilateral nerve conduction study for the common peroneal, posterior tibial, and sural nerves was performed after the proper cleaning of skin and accurate placement of electrodes. NCS was generally well tolerated by children and adolescents. In children, nerve conduction parameters reach adult values around age 3-5 years [22]. For all motor and sensory NCS, the ground electrode was placed between the stimulating and recording electrodes, whereas the stimulating and recording electrodes for each nerve were placed in their standard sites [11]. Recording from sural nerve (using antidromic method), an active recording electrode was placed behind the lateral malleolus, a reference recording electrode was placed 3 cm distal to the active recording electrode, and the stimulating electrode was located in the mid-calf 14 cm proximal to the active recording electrode. Regarding common peroneal nerve, the active recording electrode was placed over the extensor digitorum brevis muscle, the reference recording electrode was placed over the fifth toe, and the stimulating electrode had two stimulating points: the distal one was placed over the dorsal aspect of the distal lower leg between the tendon of the tibialis anterior (medially) and the extensor hallucis (laterally), and the proximal one was placed 3-4 centimeters distal to the proximal tip of the fibular head. Finally, with regard to the posterior tibial nerve, the active recording electrode was placed over the abductor hallucis whereas the reference recording electrode was placed over the big toe and the stimulating electrode was placed slightly posterior to the medial malleolus (for the distal point) and was placed over the middle of the popliteal crease (for the proximal one).

The study was approved by the ethical boards of the Faculty of Medicine, University of Khartoum, and the Sudan Childhood Diabetes Center. Written informed consents to participate were taken from the caregivers of the children. Any participant had the right to leave the study at any time. Confidentiality and patient welfare were highly considered.

Statistical analysis was performed using the Statistical Package for Social Science program (IBM SPSS) version 23. Descriptive statistic was performed for the NCS parameters to measure the dispersion of data in the form of mean, SD, minimum, and maximum. Also, the 3rd percentile of NCS parameters of the control group was considered as lower limit (cut off) of normal (reference values) so as to identify abnormal parameters in the patients' group. Transformation of patients' continuous variables into categorical variables was made based on the cut off values of the control group. Finally, the frequency of distribution of the abnormalities in the form of percentages was presented in bar charts.

## 3. Results

A total number of 50 children with type 1 DM completed the study. The mean age for the patients was 15.00 ± 2.19. The patients had a mean disease duration of 4.92 ± 3.84 years, mean age of disease onset of 10.21 ± 3.93 years, and mean HbA_1c_ of 11.28 ± 2.75. The mean age for the 26 healthy control subjects was 13.88 ± 2.46.

We used our own control values as cutoff reference values to classify NCS parameters into normal and abnormal. The values (mean ± SD) of NCS parameters of the common peroneal nerve for the control and patients, respectively, were 4.57 ± 1.67 and 3.73 ± 1.6 millivolt for the distal amplitude and 53.5 ± 4.64 and 42.39 ± 8.12 meter/second for the conduction velocity. The posterior tibial nerve values for the control and patients, respectively, were 10.67 ± 4.9 and 8.03 ± 3.81 millivolt for the distal amplitude and 50.34 ± 4.35 and 41.65 ± 5.61 meter/second for the conduction velocity. The sural nerve values for the control and patients, respectively, were 20.8 ± 9.02 and 13.95 ± 7.26 microvolt for the amplitude and 55.16 ± 6.8 and 49.7 ± 8.6 meter/second for the conduction velocity. We considered the patient's parameters (amplitude and velocity) to be abnormal if it is below the third percentile value of the control [23], which were 1.6 mV and 46.7 m/sec for the common peroneal nerve, 1.9 mV and 42.1 m/sec for the posterior tibial, and 5.7 *μ*V and 44.5 m/sec for the sural nerve. Furthermore, neuropathy diagnosis was considered if at least two abnormal parameters of NCS were present in any of the three nerves (sural, common peroneal, or posterior tibial). Nerve conduction abnormalities were detected in 44/50 (88%) of the patients, 38/50 (76%) of whom were classified as subclinical and only 6/50 (12%) as clinical neuropathy with variable clinical manifestations. Three patients presented with pure sensory symptoms (in the form of pain, numbness, and tingling), and one patient revealed in addition to the sensory symptoms motor signs (in the form of reduced ankle reflexes), while two patients showed pure motor signs (hypotonia and hyporeflexia).

**Figure 1:**
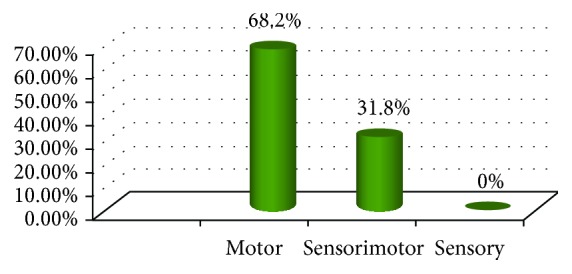
The frequency of nerve type involvement in children with DPN. The most frequent was the motor type followed by sensorimotor and no pure sensory nerve involvement (^∗^number of children with diabetic neuropathy is 44 patients).

With regard to the pattern of nerve involvement in the 44 DPN patients, it was shown that 30/44 (68.2%) revealed motor nerve involvement and 14/44 (31.8%) revealed sensorimotor nerve involvement, while none of them (0%) showed pure sensory nerve involvement as illustrated in Figure 1.

**Figure 2:**
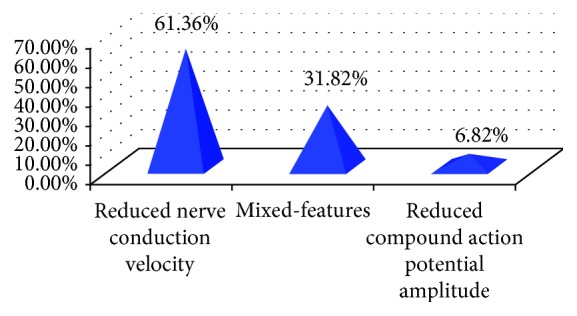
The frequency of the abnormal neurophysiological pattern in children with DPN. The most frequent feature was the reduced nerve conduction velocity, and the least was the reduced compound action potential amplitude (^∗^number of children with diabetic neuropathy is 44 patients).

From a pathological point of view, analysis of the 44 DPN patients showed that the reduced conduction velocity favoring demyelination process was the most prevalent feature in 27/44 (61.4%) and only 3/44 (6.8%) showed reduced compound potential amplitude suggesting axonal degeneration process, while the rest 14/44 (31.8%) showed mixed features (decreased velocity and amplitude together) as shown in Figure 2.

In terms of NCS parameters, the most affected was the velocity of common peroneal nerve (73.9%), followed consecutively by velocity of tibial nerve (60.2%), distal amplitude of tibial nerve (22.7%), distal amplitude of common peroneal nerve (14.8%), velocity of sural nerve (12.5%), and sural nerve amplitude (11.4%).  Table 1 showed that the most frequently affected nerve (in terms of reduced conduction velocity and/or reduced distal amplitude) was the common peroneal followed by posterior tibial and the least was the sural nerve.

## 4. Discussion

Reviewing the literature did not show any consensus regarding the pattern of nerve involvement as well as the most sensitive nerve or parameter in DPN. The pattern of nerve involvement in our patients was motor neuropathy, followed by sensorimotor, but no pure sensory nerve involvement was reported. This pattern suggests that motor nerve involvement precedes sensory ones and therefore can explain why pure sensory involvement was not reported in our study. This is different from the usual concept that sensory nerves are predominantly affected in type 2 diabetes [24]. Studying 40 children with mean disease duration (4.9 ± 3.2 years) similar to our patients' duration (4.92 ± 3.84 years), Cenesiz et al. [18] revealed that 10/40 children complained of neurological symptoms and 11/40 children showed one or more neurological deficits. They reported sensorimotor neuropathy as the most prevalent type followed by motor and the least was sensory neuropathy. Their findings might be explained by the fact that they included patients with more severe disease as documented by their clinical manifestations and therefore presented with the advanced sensorimotor neuropathy rather than pure motor insult. Many studies supported our finding that the changes in motor nerves were more frequent than changes in sensory nerves [15, 19, 20, 25]. In addition, Dyck et al. in the cohort of Rochester Diabetic Neuropathy Study found that the six most sensitive parameters of NCS in decreasing frequencies were fibular motor nerve conduction velocity, sural sensory nerve action potential, tibial motor nerve conduction velocity, ulnar motor nerve conduction velocity, tibial F-wave latency, and ulnar F-wave latency [26]. On the other hand, few studies supported the finding that sensory potential was the most sensitive indicator of subclinical involvement and concluded that sensory nerve fibers are affected before the motor ones during the course of the disease [27, 28]. Being thinner and longer, sensory nerves are more vulnerable to metabolic alterations [28].

Regarding our study, the dominance of reduced conduction velocity feature over the reduced compound potential amplitude was consistent with the finding that segmental demyelination and remyelination is the predominant histological abnormality in diabetic neuropathy [27, 29]. Contrary, a recent study (2016) carried out in 40 diabetic children with mean duration of 6.63 ± 0.25 years revealed that axonal degeneration neuropathy was the most frequent type [28].

In this study, the nerve most likely to show abnormalities was the motor common peroneal nerve followed by posterior tibial nerve and the least was the sensory sural nerve. This result conforms to other study finding that the most sensitive nerve is the motor peroneal nerve. On studying 161 diabetic children and adolescents, Kaar et al. reported that the peroneal motor conduction velocity was greatly impaired and concluded that motor conduction velocity determination of the peroneal nerve can be used in both revealing and following the abnormality in peripheral nervous function in diabetic children [30]. Hyllienmark et al. revealed that 57% out of 75 young insulin-dependent diabetic patients had abnormal conduction, especially the peroneal followed by the motor median nerves [19]. Again, a Korean study by Lee et al. on 37 patients (age 3-19 years) with newly diagnosed insulin-dependent DM reported that the most common abnormal parameters at the diagnosis were conduction velocities of motor peroneal and sensory sural nerves [20]. In addition, for the prevalence of diabetic neuropathy, Weisman and colleagues revealed that the best determinants were threshold values for peroneal conduction velocity and sural amplitude potential [31]. As well as being easy and sensitive, peroneal motor nerve conduction velocity was a good predictor of diabetic control [4]. Studying tibial, sural, and ulnar, Hendriksen et al. revealed that nerve conduction abnormalities were most pronounced in motor nerves of the leg (tibial motor conduction velocity), followed, in the order of severity, by sensory nerves of the leg (sural sensory conduction velocity), sensory nerves of the arm (ulnar sensory conduction velocity), and motor nerves of the arm (ulnar motor conduction velocity) [32].

Contrary to our finding that the prevalent abnormality was the involvement of the motor lower limb nerves, some studies reported higher sensitivity for sensory lower limb nerves or even upper limb nerves. Upon studying 30 DM1 patients, Karsidag et al. stated that the most affected nerves sequentially were the sural, peroneal, posterior tibial, median motor nerve, ulnar motor nerve, median sensory nerve, and ulnar sensory nerve [33]. Turgut and his colleagues suggested the use of the dorsal sural nerve as the best one for screening diabetic children [3]. Again, a Brazilian study evaluated the prevalence of diabetic polyneuropathy in 48 type 1 diabetics and reported that the most prevalent change was the reduction in median motor conduction velocity, followed by the fibular nerve and finally sensory conduction velocity of the sural nerve [15]. Claus et al. on studying 101 nondiabetic adults and 27 adults with type 1 DM concluded that the conduction velocities of motor median nerve and sensory sural nerve were the most sensitive parameters to distinguish normal from abnormal nerve function scores [34].

## 5. Conclusions

DPN represents a cause of major morbidity among diabetics; early detection of neuropathy using NCS helps in the prevention of its long-term complications. Defining the pattern of nerve involvement in diabetic neuropathy enables clinicians to set a protocol for screening patients with subclinical diabetic neuropathy and adopt a strict protocol for early management of children with type 1 DM.

## Figures and Tables

**Table 1:** The frequency of the affected right and left lower limb nerves.

Nerve	% of abnormal NCS parameters (velocity and/or amplitude) in the right lower limb	% of abnormal NCS parameters (velocity and/or amplitude) in the left lower limb
Common peroneal	72.7%	84.1%
Posterior tibial	61.4%	75%
Sural	22.7%	20.5%

## Data Availability

The data used to support the findings of this study are available from the corresponding author upon request.
